# Development of an Amperometric Glucose Biosensor Based on the Immobilization of Glucose Oxidase on the Se-MCM-41 Mesoporous Composite

**DOI:** 10.1155/2018/2687341

**Published:** 2018-05-10

**Authors:** Sabriye Yusan, Mokhlesur M. Rahman, Nasir Mohamad, Tengku M. Arrif, Ahmad Zubaidi A. Latif, Mohd Aznan M. A., Wan Sani B. Wan Nik

**Affiliations:** ^1^Department of Chemistry, Faculty of Science, Universiti Teknologi Malaysia, 81310 Johor Bahru, Johor, Malaysia; ^2^Institute of Nuclear Science, Ege University, Bornova, 35100 Izmir, Turkey; ^3^Institute for Community Development & Quality of Life (i-CODE), Universiti Sultan Zainal Abidin, 21300 Kuala Nerus, Terengganu, Malaysia; ^4^Faculty of Medicine, Universiti Sultan Zainal Abidin, 21300 Kuala Nerus, Terengganu, Malaysia; ^5^Faculty of Medicine, International Islamic University Malaysia, 25200 Kuantan, Malaysia; ^6^School of Ocean Engineering, Universiti Malaysia Terengganu, 21300 Kuala Nerus, Terengganu, Malaysia

## Abstract

A new bioenzymatic glucose biosensor for selective and sensitive detection of glucose was developed by the immobilization of glucose oxidase (GOD) onto selenium nanoparticle-mesoporous silica composite (MCM-41) matrix and then prepared as a carbon paste electrode (CPE). Cyclic voltammetry was employed to probe the catalytic behavior of the biosensor. A linear calibration plot is obtained over a wide concentration range of glucose from 1 × 10^−5^ to 2 × 10^−3^ M. Under optimal conditions, the biosensor exhibits high sensitivity (0.34 *µ*A·mM^−1^), low detection limit (1 × 10^−4^ M), high affinity to glucose (K_m_ = 0.02 mM), and also good reproducibility (R.S.D. 2.8%, *n*=10) and a stability of about ten days when stored dry at +4°C. Besides, the effects of pH value, scan rate, mediator effects on the glucose current, and electroactive interference of the biosensor were also discussed. As a result, the biosensor exhibited an excellent electrocatalytic response to glucose as well as unique stability and reproducibility.

## 1. Introduction

The determination of the glucose level is significant in chemical samples, biological and clinical, as well as in food processing and fermentation [1]. Diabetes mellitus especially is one of the principal causes of demise and disability in the world and is highly responsible for kidney failure, heart disease, and sightlessness. About 200 million people in the world are afflicted with diabetes mellitus [2]. So the determination of blood glucose levels rapidly, conveniently, precisely, and economically is vital for its diagnosis and effective management [3]. Therefore, the development and fabrication of a cost-effective, simple, accurate, portable, and rapid sensor for glucose are socially crucial for diabetes mellitus [4]. For this aim, electrochemical biosensors have been applied successfully for the determination of glucose.

Glucose oxidase (GOD) has been significantly used to check blood glucose levels in diabetes patients mainly due to it being inexpensive, stable, and of practical use [5] and its catalytic ability to glucose. It has a flavin adenine dinucleotide (FAD) redox point, which is entirely surrounded in the apoenzyme. It acts upon by catalysis transfer of the electron from glucose to gluconolactone. The space among its two FAD/FADH_2_ midpoints and surface the electrode is so long that explicit transfer of the electron from the enzyme to the electrode is tricky to be realized [6]. Most glucose measurements are based on the immobilization of glucose oxidase (GOD) for detecting H_2_O_2_ concentration which is obtained from the GOD enzymatic reaction [7, 8] as illustrated by the following steps:

(1) FGOXAD+glucose→FGOXADH2+gluconolactone  enzymaticFGOXADH2→FGOXAD+2e+2H+electrochemicalFGOXADH2+O2→FGOXAD+H2O2⁡enzymaticFGOXAD+2e+2H+→FGOXADH2⁡electrochemical

There are many methods for the GOD immobilization such as covalent cross-linking [9–11], electrochemical polymerization [12], sol-gel encapsulating [13], and adsorption methods [14]. Among these immobilization methods, adsorption is the simplest. Adsorption can retain the bioactivities of the immobilized enzyme well because their action needs no chemical reagents. Biosensors based on the adsorption of protein, however, are limited by the amount of the immobilized enzyme on the electrode surface and are therefore unstable. The sensitivity of the biosensor is directly proportional to the enzyme surface density on the electrode; hence, an increase in enzyme loading on the biosensor surface may result in a significant improvement in its sensitivity [15]. But it is well known that the direct electron communication between the protein and the electrode transducer is difficult because the redox sites of the protein are deeply seated in the protein shell. In order to improve the direct electrochemistry of the redox protein, nanomaterials, such as carbon nanotubes [4, 5, 16, 17], metal nanoparticles (gold, iron, nickel, etc.) [2, 18–20], ZnO nanotubes [21], platinum or sputtered platinum [22, 23], glassy carbon [24], and CdS nanoparticles [25], have been used widely due to their catalytic ability and good biocompatibility properties. A comparison of the current market glucose sensor results with published articles is given in Table 1 [26–28]. Nanoparticles can offer many advantages, such as large surface-to-volume ratio, high surface reaction activity, and strong adsorption ability to immobilize the desired biomolecules. Many metal and semiconductor nanoparticles have been chosen to prepare modified electrodes [29]. Nanomaterials, which were used as a carrier in enzyme sensor, not only increase the stability and the amount of the immobilized enzyme but also improve the catalytic activity of the protein and the responsibility of the sensor [18].

Also newly, a sequence of inorganic porous materials such as mud [30], montmorillonite [31], and mesoporous silicate [6, 32–34] has been proven to be certifying as the immobilization patterns because of their high chemical, mechanical, and thermal solidity as well as good adsorption due to its large specific surface area and absorbency. So the combination of mesoporous molecular sieves into redox enzymes could deliver a bioactive compound [6].

In this paper, we describe a simple route to the production of the Se-MCM-41 mesoporous electrode. The preparation method is simple, and selenium nanoparticles may be readily formed on a carbon paste electrode surface constructing a simple, economical, and accurate amperometric sensor for glucose. The electrode current response of glucose shows excellent stability and reproducibility. The prepared sensors can be used for the determination of glucose in human blood.

## 2. Experimental

### 2.1. Materials

Glucose oxidase (GOD from *Aspergillus niger*, E. C. 1.1.3.4), glutaraldehyde (50 wt. %), and glucose were purchased from Sigma. Ferrocene (98%, Merck) was used as received. All other reagents were of analytical grade and used without further purification. Stock solutions of glucose were prepared in 0.05 M phosphate buffer (pH 7.0) and mutarotated for at least 24 h before use. All solutions were prepared and made up with double-distilled water (DDW). Carbon graphite powder (Ultra F, 200 meshes, Johnson Matthey) and paraffin oil (from Fluka) has been used for the preparation of carbon paste electrode (CPE).

### 2.2. Preparation of Se-MCM-41/GOD Particles

The composite material was added in the flask containing 25 mL glutaraldehyde solution (5% in 0.05 M phosphate buffer, pH 6.62). The container containing the mixture of reactants was stirred at room temperature for three hours. The solution was then filtered and washed several times with deionized water to remove excess glutaraldehyde. Tollens' reagent was used to test the presence of glutaraldehyde. For this purpose, Tollens' reagent (the silver mirror test) test was carried out until a colorless solution is produced, thus indicating the absence of excess glutaraldehyde.

The glutaraldehyde cross-linked Se-MCM-41 mesopore composite has mixed with 3 mL of 2 mg·mL^−1^ GOD solution in a phosphate buffer. The reaction mixture was incubated at 0–5°C with shaking (100 rpm) for six hours. The supernatant was removed, and the composite was washed three times with phosphate buffer solution (pH 6, 0.05 M). The GOD immobilized material was recovered from the solution and stored at +4°C for subsequent uses.

### 2.3. GOD Activity Assay

Activity assay of the enzyme was carried out using *o*-dianisidine dye. The colorless dye in the reduced form reacts in the presence of peroxidase (POD) and oxidizing agent to give a brown colored solution, measurable at 500 nm using UV-Vis [35]. The GOD catalyzes the oxidation of glucose to *δ*-gluconolactone, producing H_2_O_2_ which is then measured indirectly by the oxidation of the dye in the presence of POD, following the reaction as shown in (2) and (3). The specific activity of glucose oxidase was calculated according to (2), and it was found that the 0.116 U/mg solid produced is higher than the one reported previously [11]:

(2) $$\begin{array}{c} \beta \text{-D-glucose}+O_{2}+H_{2}O\overset{\text{GOD}}{⟶}\delta \text{-glucono-1},\text{5-lactone}+H_{2}O_{2} \end{array}$$

(3) $$\begin{array}{c} H_{2}O_{2}+o\text{-dianisidine}\left(\text{reduced}\right)\overset{\text{POD}}{⟶}o\text{-dianisidine}\left(\text{oxidized}\right) \end{array}$$

(4) $$\begin{array}{c} \beta \text{-D-glucose}+O_{2}+H_{2}O\overset{\text{GOD}}{⟶}\delta \text{-glucono-1},\text{5-lactone}+H_{2}O_{2} \end{array}$$

The amount of enzyme is calculated using the following formula: enzyme (units/ml)  = (Δ*A*_500 nm_/min test − Δ*A*_500 nm_/min blank) (3.1)·(df)/(7.5)·(0.1), where 3.1 = volume (in milliliters) of assay; df = dilution factor; 7.5 = millimolar extinction coefficient of oxidized *o*-dianisidine at 500 nm; 0.1 = volume (in milliliters) of enzyme used.

### 2.4. Electrode Preparation and Electrochemical Instrumentation

The electrochemical behavior of the Se-MCM-41-GOx mesopore composite sample was studied using a working electrode made of carbon paste (CPE) modified with the solid particles. This electrode was prepared by mixing 5 mg of the obtained composite with 50 mg of graphite (1 : 10). After adding a few drops of paraffin oil, the mixture was homogenized using a pestle in an agate mortar. Then, the combination was housed in a polyethylene tube (inner diameter 2.4 mm) and polished on a smooth paper layer before conducting each of the experiments. An electric contact was made by a copper wire through the back of the electrode.

Electrochemical experiments were carried out in a conventional three-electrode cell comprising the modified carbon paste as the working electrode, an Ag/AgCl/(KCl 3 M) reference electrode, and a platinum wire as the auxiliary electrode. Experiments of all electrochemical were done with 757 VA Computrace (Metrohm) using a potential scan of −1400 mV to +1400 mV to fit the window.

All experiments performed at room temperature in 0.05 M (pH 7.0) phosphate buffer as the supporting electrolyte. Electrolyte solutions were deoxygenated with nitrogen bubbling for at least 5 min and a nitrogen atmosphere kept over the solution during electrochemical measurements.

## 3. Results and Discussion

### 3.1. Cyclic Voltammograms of GOx to the Se-MCM-41 Composite

**Figure 1:**
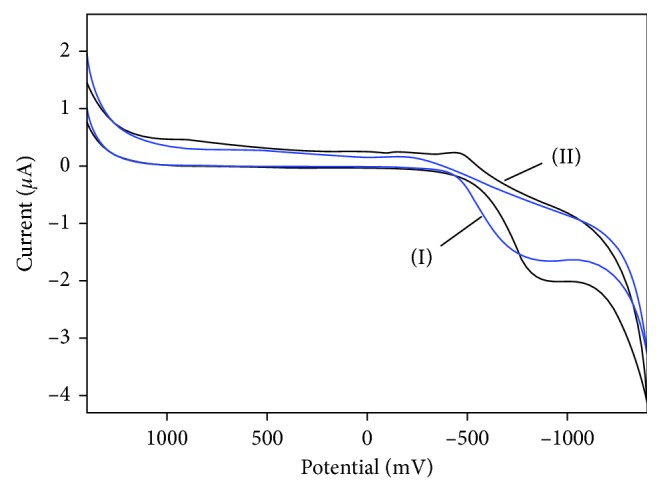
Cyclic voltammogram of (I) Se-MCM-41 and (II) immobilized GOD to the Se-MCM-41 in 0.05 M phosphate buffer (pH 7) at a scan rate of 20 mV·s^−1^.

To verify the electrochemical properties of the Se-MCM-41/GOD/CPE, we carried out the direct electrochemical measurement of the immobilized GOD on the Se-MCM-41/CPE using typical CPE electrodes without GOD.  Figure 1 displays the typical cyclic voltammograms (CV) for Se-MCM-41/CPE (I) and Se-MCM-41/GOD/CPE (II) in 0.05 M deoxygenated phosphate buffer solution (pH 7.0) over the potential range from 1.4 to −1.4 V at 20 mV·s^−1^ scan rate. As seen from the plot (I), no redox peaks were observed using the typical CPE electrode which indicated Se-MCM-41 as being electroinactive in the potential window. Plot II shows the CV of GOD immobilized on Se-MCM-41/CPE, and a pair of oxidation-reduction peak appears. The results indicated that the reaction of catalytic oxidation of glucose by GOD occurred and the immobilized GOD retained its electrocatalytic activity for the oxidation of glucose [36, 37].

Flavin adenine dinucleotide is a part of the GOD molecule and is known to undergo a redox reaction, where two electrons and two protons were exchanged, and the electrochemical response of GOD immobilized on the solid surface is due to the redox reaction of FAD [38]. The FAD redox potentials in various enzymes ranged from 190 to −490 mV. In plot (II), the FAD peak appeared about −470 mV, which was acceptable, and suggested that GOD bound to the Se-MCM-41 composite was active in the electron transfer [11].

The main cause for the transfer electron between the silica-based electrode and the enzyme might be due to the electrostatic relations, such as hydrogen bonding and hydrophilic attraction between MCM-41 and glucose oxides (GOD). The communication between MCM-41 and GOD is much stronger than that between CPE and GOD due to the presence of Si-OH groups on the external surface of MCM-41. In our study, the glucose oxides (GOD) should be immobilized on the external surface of Se-MCM-41 by physical adsorption because the pore diameter of GOD (about 4–6 nm) is bigger than the size of Se-MCM-41 (about 2.7 nm) [6]. Due to the presence of many acidic silicon hydroxyl (Si-OH) groups on the outer surface of the Se-MCM-41, the oxidation reaction of GOD will be not easy thermodynamically.

### 3.2. Effect of Scan Rate

**Figure 2:**
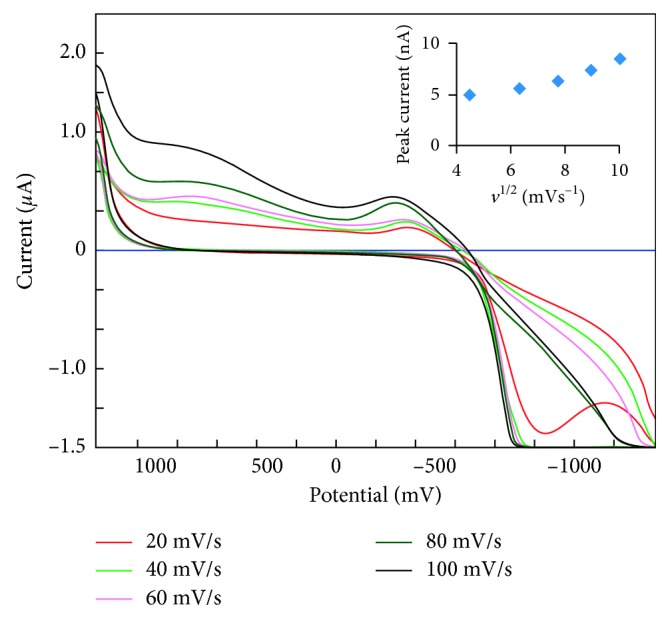
Cyclic voltammograms of Se-MCM-41/GOD/CPE in pH 7.0 PBS at 20, 40, 60, 80, and 100 mV·s^−1^ scan rates (from inner to outer). Inset: peak currents of plots versus *v*^1/2^.

The effect of scan rate on the electrochemistry of the immobilized GOD is shown in Figure 2. With increasing scan rate, the anodic peak currents of the GOD increased linearly, and the anodic peak potential of GOD is shifted to a more positive value. The dependence of the current (*I*_p_) on the square root of the scan rate (*v*^1/2^) (inset in Figure 2) is an essential diagnostic criterion for establishing the type of reaction mechanism by cyclic voltammetry. It suggests that the Se-MCM-41 composite is sufficiently thick and the electron transfer between selenium MCM-41 composite is slow, leading to the system to mimic semi-infinite linear diffusion [39].

### 3.3. Effect of pH on the Peak Current

**Figure 3:**
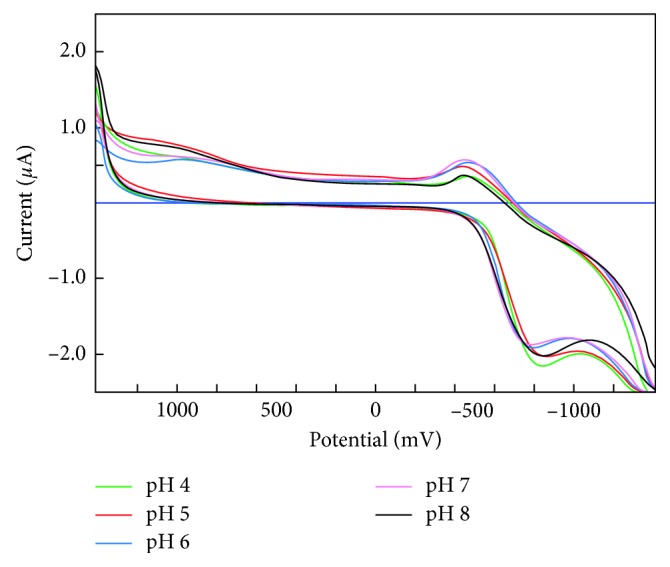
Effect of pH on the response of the biosensor (Se-MCM-41/GOD/CPE) in the presence of 2 mM glucose.

The effect of pH on the anodic peak current was investigated over the range pH 4.0–8.0 (buffer solution of K_2_HPO_4_–KH_2_PO_4_) in the presence of 2 mM glucose at 25°C. The resulting *I* versus pH (4–8) data are illustrated in Figure 3. As can be seen, the peak potential of GOD is dependent on the solution pH with the maximum response observed at pH 7.0, which is consistent with that of most GOD-based glucose biosensors. Therefore, we fixed the solution pH at 7.0 for the investigation of the analytical performance of Se-MCM-41 carbon paste electrode covered with GOD. Either the strongly acidic solution or alkaline solution would decrease the bioactivity of the enzyme. Thus, the proposed biosensor was practical to detect glucose levels in real samples at neutral pH value [40]. These results are similar to previously reported values of optimum pH for the use of glucose oxidase with other artificial electron acceptors [5, 29, 41–43].

### 3.4. Effect of the Glucose Concentration

**Figure 4:**
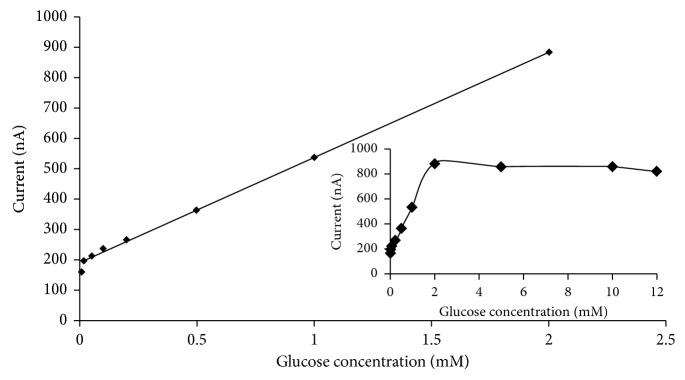
Correlation between the maximum current and the concentration of glucose.

The cyclic voltammograms of Se-MCM-41/GOD/CPE with successive addition of glucose to air-saturated 0.05 M PBS (pH 7.0) are shown in Figure 4. The peak current of Se-MCM-41/GOD/CPE increased linearly with increasing concentration of glucose up to 2 mM. The calibration range of glucose concentration was prepared from 0.01 to 12.0 mM. The linear response range of the sensor to glucose concentration was from 0.01 to 2.0 mM with a correlation coefficient of 0.997. The sensitivity of Se-MCM-41/GOD/CPE to glucose was found to be 0.34 *μ*A·mM^−1^, which is higher than values found in the literature for GOx/PtNPs/CNTs/carbon paste (0.28 *μ*A·mM^−1^), Nafion/GOx film electrode (51.0 nA·mM^−1^), and poly(*o*-phenylenediamine) covered screen-printed electrode (16.6 nA·mM^−1^) [44–47].

According to the Lineweaver–Burk form of the Michaelis–Menten equation, the relation between the reciprocal of the response current and the reciprocal of glucose concentration can be obtained. The apparent Michaelis–Menten constant (*K*_*m*_), an indicator of enzyme-substrate reaction kinetics, can be used to evaluate the biological activity of the immobilized enzyme, and this constant can be calculated from the Lineweaver–Burk equation, given below:

(5) $$\begin{array}{c} \frac{1}{I_{\text{ss}}}=\left(\frac{K_{m}}{I_{\max}}\right)\;\;\left(\frac{1}{C_{g}}\right)+\frac{1}{I_{\max}}, \end{array}$$

where *C*_*g*_ is the substrate concentration, *I*_ss_ is the steady-state current, and *I*_max_ is the maximum current measured under substrate saturation [48].

Here, Michaelis–Menten constant *K*_*m*_ and the maximum response current of the biosensor based on the Se-MCM-41/GOD electrode are calculated and equal to 0.02 mM and 0.45 *µ*A, respectively. The low *K*_*m*_ value of 0.02 mM indicates the affinity of the enzyme to the electrode, which was smaller than those of 5.20, 22.8, 14.4, 21, and 37.6 mM, for immobilized GOD [5, 45–48], indicating that the GOD immobilized on Se-MCM-41/GOD has high affinity to glucose. These results imply that the biosensor based on the Se-MCM-41/GOD electrode is valuable and sensitive.

### 3.5. Glucose Bioelectrocatalytic Oxidation

**Figure 5:**
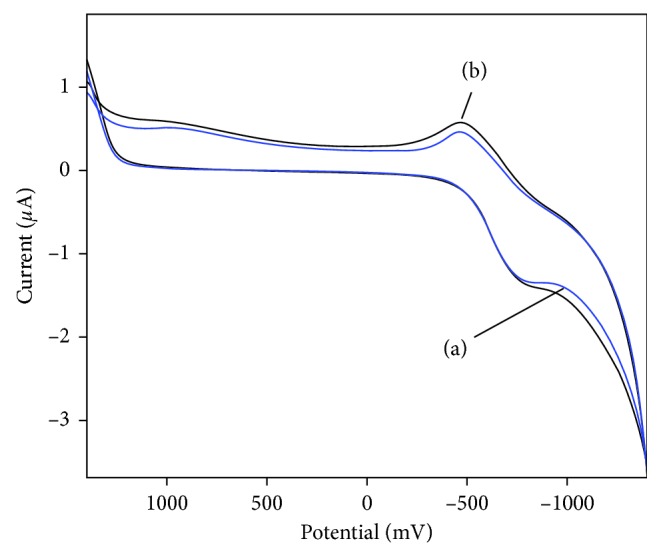
Cyclic voltammograms of Se-MCM-41/GOD/CPE in 0.05 M PBS (pH 7.0) containing 0.2 mM ferrocene in the absence (a) and presence (b) of 2.0 mM glucose.

Although the direct transfer electron of GOD was achieved, some intermediaries are still used to accelerate the electron transfer rate between electrode and GOD [8, 9]. To increase the response current, ferrocene, a satisfactory electron transfer mediator has been used in the biosensor based on Se-MCM-41/GOD/CPE [44].  Figure 5 has shown cyclic voltammograms of the Se-MCM-41/GOD/CPE in N_2_-saturated, pH 7.0 PBS containing 0.2 mM ferrocene as the facilitator. When 2.0 mM glucose was added to the solution, the anodic peak current increased, using ferrocene as the mediator in N_2_-saturated solutions, consequently, demonstrating that Se-MCM-41/GOD/CPE is able to electrocatalyze the oxidation of glucose. These results can be explained from the following equations:

(6) $$\begin{array}{c} \begin{array}{l} \text{Glucose}+\text{GOD}\left(\text{ox}\right)\to \text{gluconolactone}+\text{GOD}\left(\text{red}\right)\\ \text{GOD}\left(\text{red}\right)+2\;\;\mathrm{ferrocene}\to \text{GOD}\left(\text{ox}\right)+2\;\;\mathrm{ferrocene}+2H^{+}\\ 2\;\;\mathrm{ferrocene}\to 2\;\;\mathrm{ferrocene}+2e^{-} \end{array} \end{array}$$

### 3.6. Analytical Performance

The amperometric response characteristics of the enzyme electrode are affected by the electroactive interferents. In this section, the effects of these factors on the behavior of the GOD electrode, reproducibility, and stability have been investigated in detail and discussed accordingly.

#### 3.6.1. Reproducibility

The reproducibility of the glucose biosensor was investigated by successively detecting 0.10 mM glucose ten times; the relative standard deviation (RSD) calculated was 2.8%. The RSD for the detection of 1.0 mM glucose with three sensors prepared independently under the same conditions was 3.6%, demonstrating a good reproducibility of the measurements performed.

#### 3.6.2. Stability

The stability of the enzyme electrode was also investigated by amperometric measurements. It retained 91% of its initial current response, when the enzyme electrode was stored at +4°C for glucose after intermittent use over a 10 days interval. So, it can be presumed that the presence of nanoparticle selenium incorporated MCM-41 is very efficient in retaining the enzyme activity of GOD.

#### 3.6.3. Interferences

The effects of interference have been observed by testing the amperometric response of 0.1 mM glucose in the presence of their normal physiological concentration (0.1 mM for ascorbic acid and 0.1 mM for citric acid) [48]. When the glucose-to-interferent concentration ratio was 1 : 1, a decrease of 14% for ascorbic acid or an increase of 25% for citric acid in the current value of the 0.5 mM glucose was observed, respectively. In addition, when the ratio of glucose to interferent was 1 : 2 for the ascorbic acid or citric acid, the response current decreased significantly as compared to the response for the 1 : 1 ratio. These results suggested that the presence of interferences such as ascorbic or citric acid did not affect the current measurement of the GOD significantly using the Se-MCM-41/GOD. The results also suggested that the proposed glucose has high selectivity and compatibility on the Se-MCM-41/GOD as the support material for the sensor.

## 4. Conclusion

The preparation of a new type of glucose oxidase electrode using nanoselenium particles and the MCM-41 mesoporous composite was well demonstrated. The preparation of the electrode is easy, fast, and reproducible. Cyclic voltammetric results confirmed that the prepared electrode presents a highly electrocatalytic activity for the oxidation of glucose. The optimum pH was obtained at pH 7.0 which is the optimal environment herein. Since the pH value of human blood was around 7.4, the potentiometric glucose biosensor was suitable for measuring the concentration of glucose in human blood. Under the optimized experimental conditions, the catalytic currents are linear to the concentrations of glucose from 1 × 10^−5^ to 2 × 10^−3^ M. The detection limit was 1 × 10^−4^ M with a signal to noise ratio of 3. The sensor exhibits good reproducibility and stability during the measurement. At the same time, the biosensor demonstrates high sensitivity (0.34 *µ*A·mM^−1^). Besides, the biosensor possesses high sensitivity and excellent chemical and mechanical stability. All the results show that the prepared Se-MCM-41/GOD can provide a promising material for biosensor designs and other biological applications.

## Figures and Tables

**Table 1:** Comparison between current market CGMS/sensor results devices [26–28].

Brand	Guardian Real-Time	MiniMed 530G with Enlite	Dexcon G4 Platinum	FreeStyle
Company	Medtronic	Medtronic	Dexcom	Abbott
FDA approved date	2006	2007	2007	2008
Sensor life (d)	3	6	7	5
Sensor style	Insertion under skin	Insertion under skin	Insertion under skin	Insertion under skin
Startup initialization time (h)	2	2	2	10
Calibration (Y/N) with finger-stick test	Y, 2 h after insertion, the first 6 h, and then every 12 h	Y, 2 h after insertion, the first 6 h, and then every 12 h	Y, every 12 h	Y, approximately 1, 2, 10, 24, and 72 h after insertion

CGMS = continuous glucose monitor system; FDA = Food and Drug Administration.
